# Internationally educated nurses’ and medical graduates’ experiences of getting a license and practicing in Sweden – a qualitative interview study

**DOI:** 10.1186/s12909-018-1399-4

**Published:** 2018-12-05

**Authors:** Elisabet Eriksson, Sören Berg, Maria Engström

**Affiliations:** 1 Department of Health and Caring Sciences, University of Gävle, Kungsbäcksvägen 47, 801 76 Gävle, Sweden; 2 Department of Medicine and Health Sciences, Department of Cardiothoracic Surgery, Linköping University, County Council of Östergötland, Linköping, Sweden; 3 Department of Health and Caring Sciences, University of Gävle, Gävle, Sweden; 4 Department of Public Health and Caring Sciences, Uppsala University, Uppsala, Sweden; 5 Nursing Department, Medicine and Health College, Lishui University, Lishui, China

**Keywords:** IEN, IMG, Intercultural competence, License, Work experience

## Abstract

**Background:**

The Swedish healthcare system has an increased need for nurses and physicians, and the number of International Educated Nurses (IENs) and International Medical Graduates (IMGs) seeking job opportunities and a license to practice in Sweden is rising. This study explored how IENs and IMGs describe their experience of getting a license to practice, their perceptions of working in Sweden and of how their intercultural competence is utilized.

**Method:**

A qualitative study based on semi-structured interviews with 11 IENs and 11 IMGs. The interviews were conducted between 2015 and 2017. The data were analyzed using qualitative content analysis.

**Results:**

Three main themes were identified: ‘Getting a license – a different story,’ ‘The work is familiar, yet a lot is new,’ ‘Trying to master a new language.’ The time to obtain a license to practice and finding a job was shorter for IENs and IMGs coming from European countries than for those from non-European countries. Some of the experiences of getting a license to practice and of entering a new workplace in another country were the same for nurses and physicians. In general, both IENs and IMGs felt welcomed and used their intercultural competence at work. Lack of language skills was regarded as the main problem for both professions, while workplace introduction was shorter for IMGs than for IENs.

**Conclusions:**

Problems related to language and culture are often underestimated, therefore organizations and managers employing IENs and IMGs should provide longer workplace introduction to facilitate the acculturation process. More time-efficient language courses specifically adapted to IENs and IMGs could make the transition easier and shorten the time to obtain a license to practice for both professions.

**Electronic supplementary material:**

The online version of this article (10.1186/s12909-018-1399-4) contains supplementary material, which is available to authorized users.

## Background

Globalization has enabled increased mobility for internationally educated nurses and physicians, who comprise a significant part of the global health workforce [1, 2]. An aging population and shortages of health professionals in high-income countries, as well as an opportunity for migrants to attain better living conditions, have also affected the migration of nurses and physicians from low-income countries to high-income countries. Across 22 OECD countries, the share of foreign-born nurses increased from 11 to 14.5% between 2000 and 01 and 2010–11, and the share of foreign-born doctors increased from 19.5 to 22% (OECD 2015).

Since 2003, the Swedish National Board of Health and Welfare (SNBH) has issued more licenses to practice medicine to persons educated abroad than to persons educated in Sweden [4]. In 2015, the SNBH issued 1509 licenses to persons educated abroad and 1070 to persons educated in Sweden. The physicians educated abroad come from the EU/EAA countries, from non-EU countries or are Swedish citizens who have completed their medical education abroad, most often in another EU country [3, 4]. The latter group faces no integration or language problems. In 2013, International Medical Graduates (IMGs) made up 26% of the Swedish medical workforce, with the majority coming from Poland, Germany, Hungary and non-EU countries [4]. There has also been an increase in the number of licenses issued to International Educated Nurses (IENs). In 2015, the SNBH issued 672 licenses to persons educated abroad compared to 229 licenses in 2009 [4].

During the integration process, many IENs and IMGs face various barriers before finding a job in the host country. It can be difficult for IENs and IMGs to prove the accreditation of their nursing or medical education or to obtain required documentation from their home country [5]. Often IMGs need to prove their skills or pass medical equivalency tests before they are licensed to practice [6, 7]. Further, IENs and IMGs often have to take language courses before they can apply for a license in the host country [5, 8, 9]. For example, in April 2016, the SNBH introduced a language requirement in Swedish, Norwegian or Danish for all IMGs and IENs seeking a license to practice their profession. Prior to 2016, only IENs and IMGs from countries outside the EU/ EEA had to prove their skills in Swedish, and the employer was responsible for ensuring that physicians coming from the EU could speak a Nordic language when working. In some countries, the license application process can delay the IENs’ or IMGs’ employment from some months up to several years [5]. For some IENs, this process becomes so protracted and demanding that they never apply for a nursing license in the host country, but instead work in unlicensed professions [8].

Once licensed to work in their profession, IENs and IMGs encounter many challenges when starting to work in the new country. Previous literature from the US, Canada, Australia and the UK – the major recipient countries – has described the challenges for IENs and IMGs and focused on the need to learn and communicate in a new language [8, 10–17], the challenge of the physician-patient relationship [18, 19], the nurse-patient relationship [20], adjusting to a new healthcare system [8, 14, 18], adjusting to more independence, assuming a leadership role [8, 11, 15, 18, 21] and handling newer technologies [10].

Apart from adjusting to a new healthcare system, IENs and IMGs also have to learn about the culture in the host country [22–24]. When building their lives in the new country, some IENs and IMGs describe the differences between countries as culture shock [10, 25–27]. However, moving across cultures also influences cultural knowledge and skills among IENs and IMGs. Although the meaning of intercultural competence is not homogenous, a definition from the US is used in the present paper: one’s ability to communicate effectively and appropriately in intercultural situations based on one’s intercultural knowledge, skills, and attitudes’ ([28] p. 248). Furthermore, studies have reported that IENs and IMGs experience discrimination in the workplace in the host country [17, 29]. Discrimination, marginalization or social isolation of IENs can come from patients, visitors, colleagues and supervisors [2, 8, 26, 30, 31].

Although extensive work has been done to explore the challenges that IENs and IMGs face in the major recipient countries, a wider perspective from Europe is needed. Studies from countries with less immigration will add to our understanding of how IENs and IMGs adjust to the workplace and the culture in the host country.

The aim of the present study was to describe IENs’ and IMGs’ experiences of getting a license to practice and work in the Swedish health and social care system. A further aim was to evaluate their ability to use their intercultural competence at work, and whether intercultural competence could be an asset in an increasingly multicultural society.

## Method

### Design

The study was descriptive in design and took a qualitative approach, as its aim was to gain an understanding of individuals’ experiences [32]. Because both the subject and the context were of importance, the method of content analysis was used to analyze the data [33], where the individual interview is the unit of analysis. The study was based on semi-structured interviews with 11 IENs and 11 IMGs.

### Settings and participants

Nurses who have received their education in another EU or EEA Member State and who wish to work in Sweden as a registered nurse responsible for general care need formal recognition of their professional qualifications. For nurses educated outside the EU and EEA, there are three routes for supplementing one’s training before applying for a license in Sweden. The first route is through the National Board of Health and Welfare (Table 1). The second route is to complete an additional training program at a college or university and then apply for a Swedish license. This is a one-year full-time program including clinical training. The third route is to go to school to obtain a Swedish degree after three years of studies and then apply for a license. This route is mainly for nurses who do not have a post-secondary degree or whose education is not equivalent to the system in Sweden.

**Table 1:** A simplified description of the application process via the proficiency test by the National board of health and welfare for nurses and doctors

Nurses		Doctors	
Educated within the EU or EEA^a^ *Through the National Board of Health and Welfare (applies from September 2016)*	Educated outside the EU and EEA^a^ *Through the National Board of Health and Welfare (applies from September 2016)*	Educated within the EU or EEA^a^ *Through the National Board of Health and Welfare (applies from September 2016)*	Educated outside the EU and EEA^a^ *Through the National Board of Health and Welfare (applies from July 2016)*
1. Prove formal recognition of professional qualifications. Prove a certificate which confirms that the applicant meets one of the following articles in EU directive 2005/36/EC (articles, 23,31, 33, 33a)	1. Apply for an assessment of foreign education	1. Prove formal recognition of the professional qualifications. Prove a certificate which confirms that the applicant meets one of the following articles in EU directive 2005/36/EC (articles 23,24)	1. Apply for an assessment of foreign education
2. Show certified knowledge of Swedish, Norwegian or Danish language	2. Learn Swedish	2. Show certified knowledge of Swedish, Norwegian or Danish language	2. Learn Swedish
3. Prove a “Certificate of Current Professional Status”	3. Pass the proficiency test in Nursing	3. Prove one of the certificates “Certificate of Good Standing” or “Certificate of Current Professional Status”	3. Pass the proficiency test in Medicine
	4. Pass a course on Swedish laws and legislation		4. Pass a course on Swedish laws and legislation
	5. Clinical training (3 months)^b^		5. Clinical training (6 months) ^b^
	6. Show certified knowledge of Swedish, Norwegian or Danish language and submit a formal application for a license to practice^c^		6. Show certified knowledge of Swedish, Norwegian or Danish language and submit a formal application for a license to practice^c^
The processing time is about 3 months	The processing time is about 2 months	The processing time is about 3 months	The processing time is about 2 months
Fee for applying for a license is SEK 700	Fee for applying for a license is SEK 700	Fee for applying for a license is SEK 2300	Fee for applying for a license is SEK 2300

^a^Including, Norway, Iceland, Liechtenstein and Switzerland

^b^The National Board of Health and Welfare does not assist in finding a place for the clinical training

^c^The formal application can only be submitted after steps 1–5 have been fulfilled

Swedish law protects the title “licensed physician,” and those who wish to practice as a doctor of medicine in Sweden need a Swedish license or special authorization. Those who wish to practice as a specialist need to first be licensed as a doctor of medicine, after which time they can take specialist training to receive a certificate. Doctors who have an education from another EU or EEA member state and wish to work in Sweden as doctors need formal recognition of their professional qualifications. Similarly to nurses, there are three routes for supplementing one’s training and obtaining a license in Sweden. The first route is through the National Board of Health and Welfare (Table 1). The second route is to take an additional training program at a university and then complete an internship before applying for a license. The third route is to go to medical school to obtain a Swedish degree followed by an internship and then apply for a license. A residence permit is needed for work longer than three months for EU/EEA members, and a residence and work permit is needed to work in Sweden for those educated outside the EU/EEA.

Performing face-to-face interviews requires geographical accessibility. For this reason, seven hospitals in southern and central Sweden were contacted. Two hospitals declined to take part in the study on nurses and one hospital in the study on doctors. Of the included hospitals, four were secondary healthcare facilities and two were tertiary healthcare facilities. A purposive sample of IENs and IMGs was selected to achieve variation in country of education, age, gender, and work experience in Sweden. The preliminary selection criterion was IENs and IMGs with two years of work experience in Sweden, although the selection criterion was revised to include all nurses and doctors educated abroad due to the difficulty involved in recruiting participants to the study. Therefore, two midwives working as auxiliary nurses were included because they had not yet passed the tests for working as midwives in Sweden. One IMG who had not passed the language test but was a trainee at a Swedish hospital was also included. Their perspectives were deemed interesting as they were in the middle of the process of obtaining a license. Human resources departments assisted with contact information for possible participants. Participants received an email and those interested in participating were encouraged to reply by email or phone. Snowball sampling was used to achieve an adequate sample size. The researchers did not know the potential population of IENs and IMGs at the selected hospitals, but given the size of the hospitals and the number included, it was expected that a sufficient number of healthcare professionals could be recruited. This was nonetheless a challenge. The Human Resources (HR) department at the hospitals had no list of the number of IENs and IMGs, which made the inclusion process challenging. To further strengthen credibility participants were selected who varied in gender, age, home country and work experience in Sweden. The first author contacted the participants prior to the interview to decide where and when the interview could be conducted; she had no prior relationship with the participants. Five IMGs may have met the second author previously during their supplementary education, but there was no teacher-student relationship at the time of the interview.

### Data collection

Semi-structured interviews were held in Swedish by the first author at a place convenient for the participant, usually a meeting room at their workplace. Interviews with IENs (face-to-face interviews: *n* = 9; telephone interviews: *n* = 2) were conducted from October 2015 to June 2016 and with IMGs (face-to-face interviews: *n* = 4; telephone interviews: *n* = 7) from May 2016 to January 2017. The interviews with the IENs lasted between 40 and 70 min (mean 50 min) and with the IMGs 49–67 min (mean 55 min). They were recorded on an MP3 player. An interview guide was used (additional file 1) with open-ended questions covering five areas: (1) work experience, (2) work satisfaction, (3) work environment, (4) career opportunities, and (5) cultural competence. The data analyzed here concern areas 1 and 5. Data on IENs’ access to structural empowerment at work have already been published [21]. The interview questions included: What introductory program did you received to adapt your working methods to a Swedish context? Tell me about the first time you worked as a nurse/doctor in Sweden, start with the first day, how was it? How was the first month? Have you noticed conflicts between nurses/doctors with foreign education and Swedish nurses/doctors or between nurses/doctors from different cultures? Although our sample included participants who had worked in Sweden for many years and those not yet licensed to practice, the participants could describe the path toward obtaining a license in detail and the experiences at their first workplace. Thus, this period of time seemed to have made a strong impression on them as individuals.

### Data analysis

All interviews (IENs and IMGs) were transcribed verbatim and analyzed using qualitative content analysis [32–34]. Interviews with the IENs were analyzed using *inductive* qualitative content analysis, while interviews with the IMGs were thereafter analyzed using *deductive* qualitative content analysis [32–34]. By both listening to and reading the transcripts, the first author gained a sense of the participants’ experiences. Then, meaning units related to the study aim were identified and extracted from the IENs’ transcripts. Thereafter, the meaning units were coded [33]. By looking for similarities and differences among the codes, groups of codes sharing common characteristics were organized into categories and labeled. The first and last author discussed the labeling of the codes and categories during this process, and as suggested by Graneheim and Lundman: ‘The intent here is not merely to verify that data are labeled and sorted in exactly the same way, but to determine whether or not various researchers and experts would agree with the way those data were labeled and sorted’ ([33] p 110). Regarding analysis of the IMGs’ interview data, the first author again listened to the interviews and read the transcripts. Subsequently, meaning units related to the study aim were identified and extracted from the IMGs’ transcripts. In this deductive part of the analysis, the meaning units were assign to pre-determined categories, those emerging from the interviews with IENs. No new categories emerged when analyzing the interviews with the IMGs, and there was no lack of data to fit the pre-determined categories from the interviews with the IENs. The final stage involved interpreting the data to generate themes, and the authors agreed on three descriptive themes [32–34]. To enhance the trustworthiness of the study, the first and last author discussed all steps during the data analysis process [33]. Furthermore, a detailed description of the participant’s demographic characteristics and the Swedish context has been presented, as well as verbatim quotes from the participants to enable readers to evaluate the authors’ interpretations. Dependability was strengthened by the authors having an open discussion about interpretation of the findings and engaging in reflective thinking. Open Code, a computer application, [35] was used to assist with data organization.

### Ethical considerations

The Regional Ethical Review Board at Uppsala University approved the study (reg. no. 2014/171). All participants received oral and written information prior to the interview. Participation was voluntary, and participants were informed that they could refrain from discussing particular questions and withdraw from the study at any time. The data were deidentified.

## Results

Background characteristics of the participants are presented in Table 2. The IENs were mainly female (*n* = 10) and had been educated within the EU (*n* = 7). Only one IEN had a postgraduate education from abroad. Their mean age was 35 years and their working experience in Sweden ranged from 0.5–19 years (mean 5.5). For IENs, the time required to receive their license varied from having a recruitment company help with obtaining a license prior to moving to Sweden, to almost four years for nurses coming from outside the EU. The IMGs were mainly male (*n* = 8) and almost half of them had been educated within the EU (*n* = 5). There were more IMGs than IENs who had a postgraduate education from abroad (*n* = 6), and two IMGs had a PhD. The IMGs were older (mean 39) than the IENs, and their working experience in Sweden was shorter (mean 2.7 years). Among the IMGs, five had not yet received a license to practice medicine in Sweden at the time of the interview. They were in a clinical training program, in the process of completing their internship or had not passed the language test. The time required to receive their license for IMGs varied, and could take up to five years. The time to being licensed varied from being recruited and having a job upon arrival to Sweden, to looking for a job themselves. One IMG described having sent up to 100 applications before finding employment, while others had established contacts with employers during their internship. The time for introduction (working alongside other health care professionals/ working under close supervision by an experienced colleague) at their first place of employment in Sweden varied, but was generally described as longer for the IENs (1.5–6 months) than for the IMGs. The IMGs began working with patients directly, but had fewer patients per day compared to native staff and, as they put it, started with “simpler” patient cases.

**Table 2:** Characteristics of IENs and IMGs

Categories	IENs *N* = 11	IMGs *N* = 11
Male (n)	1	8
Female (n)	10	3
Age years, range (mean)	(35) 25–59	(39) 31–45
Years of working experience prior to Sweden, range (mean)	(5.3)^a^ 0–17	(4.5) 0–15
Years of working experience in Sweden, range (mean)	(5.5) 0.5–19	(2.7)^b^ 0–15
Country of education	Algeria, Bosnia, Bulgaria, Germany, Great Britain, New Zealand, Poland, Serbia, Spain, Sudan	Poland, Italy, Syria, Romania, Greece, Uzbekistan, Ukraine, Colombia, Iraq
Working area in Sweden	Infection ward, medical ward, maternity care, delivery care, elderly care	Health center, dermatology department, maternity care, psychiatry, emergency department
Completed education, year	1987–2013	1981–2011
Specialization (postgraduate education from abroad)	1	6
PhD (at time of interview)	0	2
Recruited by an employer Trained within EU/EEA Trained outside EU/EEA Additional training at university (1 year) Route through the National Board of Health and Welfare	1 7 4 2^c^ 2	2 5 6 5 1

^a^Two missing values

^b^Including clinical training and internship

^c^Participated in an older training system about one year

### Themes and categories

The results of the analysis are presented as nine categories and three themes: ‘Getting a license – a different story,’ ‘The work is familiar, yet a lot is new, ’ and ‘Trying to master a new language’ (Table 3). Key themes and verbatim quotes are also presented in Table 4.

**Table 3:** Codes, categories and themes developed during the analysis of IENs’ interview transcripts

Code	Category	Theme
Easy to get a license	The short story	Getting a license – a different story
A challenge as a non-EU-member	The long story	
Accommodating	Being introduced	The work is familiar, yet a lot is new
Major differences between Sweden and the home country	Observing differences and adjusting to them	
Competent as a nurse	Feeling competent	
Understanding of other cultures	Using cultural competence and life experiences	
Attending different language courses	Learning a language in various ways	Trying to master a new language
Feel lesser worth than a Swedish colleague	Feeling insecure and inferior in relation to limited language skills	
Learning Swedish takes time	Language skills – the main problem

**Table 4:** Quotations and themes from the interviews with IENs and IMGs

Quotations	Theme
	Getting a license- a different story
*“I moved here in 2004; in 2007 I was done with the language and* supplementary training *and could start working. The path was pretty long.”* (IEN 10)	
“I came for a visit. I went to the National Board of Health and Welfare and asked them what I needed, which documents, and then they helped me. Then I got my nursing license.” (IEN 7)	
	The work is familiar, yet a lot is new
“What’s negative about… in all ways I guess, it’s the bureaucracy and well, that fact that you need much much more time for the administrative documentation even though you’re a physician” (IMG 9).	
“… and for instance, I make a schedule for myself, so I can follow that schedule so I make less and less mistakes. Well, because you have to remove… the Italian system and only think about the Swedish system” (IMG 5).	
“I think I’m academic and I think I have so many different experiences under my belt. I’m competent. I feel I’m able to manage and do a good job, I think so. It’s a combination of education and the different jobs I’ve had” (IEN 7).	
*“… I’ve passed the Swedish tests so I’ve done my internship in Sweden. So I’m approved, I have my Swedish license. I shouldn’t doubt my competence; my level of competence is equivalent to Swedish physicians’ competence”* (IMG 9).	
“Yes it was pretty stressful because there were two fronts, you could say; one was the language and the other was knowledge at my job because I lacked experience. So I’d say it was pretty difficult.” (IMG 6)	
*“Sometimes the patients who arrive who come from our countries, Syria or Lebanon or others, they say there’s a big difference, and they’re right, because we… Here you have to follow, like I said, a program or a system, but in our countries you don’t have to follow this system… so sometimes I have to explain to them, the patients, that it’s the system that’s for this way, you have to follow this system”* (IMG 5).	
	Trying to master a new language
“… studied Swedish C, then I worked as an assistant nurse here, and then, I didn’t understand a lot, but I could listen and understand more, but couldn’t express myself. It was a bit harder during that period but it was exciting. And I told the patients I worked with where I came from and I spoke pretty slowly and they helped me with the language, so I learned a lot of the language here [workplace]” (IEN 3).	
“But making suggestions, moving ahead is difficult. And I imagine this depends on language, because I speak differently, maybe my language isn’t as refined as others’. I can’t discuss things… I don’t get involved in discussions, because I know my language isn’t good enough” (IGM 2).	

### Getting a license – A different story

The first theme involves the various experiences during the process of applying for a Swedish license. The differences were related to whether the nurses and doctors came from the EU or outside the EU.

#### The short story

Nurses and midwives coming from the EU found it quite easy to obtain a Swedish license. They described the process as quite simple and straightforward. Some reported having received assistance with the application from the SNBH. One IEN first found it difficult to get a place for her clinical training, but it worked out well when she received help from the public employment service: “*I thought it would be tricky getting in because I’m not a nurse like all the others. But it went really smoothly”* (Midwife 4).

Similarly, IMGs from the EU did not describe any major difficulties regarding obtaining a Swedish license to practice medicine. They had prepared themselves in their home country to ease the process. One IMG had prepared all documents, sent them and then came to Sweden when he had obtained the license. He had already started learning Swedish and got a job quickly. One IMG had finished his specialization before applying for the license, knowing that the process would then be shorter: “*I didn’t have any problems with my documents, it just took a certain amount of administrative time”* (IMG 1).

#### The long story

Obtaining a Swedish nursing license was portrayed as a lengthy process for those coming from a country outside the EU. After passing the language tests, the IENs from outside the EU applied for the supplementary training or the proficiency test. However, the IENs sometimes did not receive information from the SNBH about what was required of them before they could apply for a Swedish license. Some did not know where and to whom they should turn to start the application process: “*I got no support. I was completely alone and went to the public employment office and didn’t have a clue about what I should do. I went around and wasted about 6 months not knowing what to do”* (IEN 9). One IEN coming from a conflict area said she could not travel back to get the certificates needed for the nursing application. The IENs reported having had a difficult time learning Swedish and taking the supplementary training or the proficiency test. Many of them also worked as auxiliary nurses to have an income alongside their studies. The studies required a great deal of effort, and the IENs reported not having enough time to both study and work: “*… and I worked a lot every summer on the infection ward. During fall and spring term I just worked a little, maybe 50% at the same time as I was studying for my knowledge text for being a nurse in Sweden*” (IEN3). Regarding the supplementary training, some IENs thought it would be better if they had more clinical practice and fewer theoretical topics.

The IMGs coming from outside the EU reported that it took a very long time before they could apply for a Swedish license. Similar to the IENs, they had to pass the language tests before continuing the process toward getting a license. Regarding the supplementary training, the IMGs reported that the course had prepared them for their work. However, some mentioned that there was a strong focus on medical theory followed by practical training and less information on how the Swedish healthcare system works. When they started work, they had limited knowledge about planning care for patients leaving the hospital, contacts between primary care and the hospital, collaboration with the municipality, writing certain certificates (e.g., for economic reimbursement) and knowing who is responsible for different areas. Further, even though the course was greatly appreciated, some described it as an intensive and tough time. One IMG failed the last examination: “*But then I missed the exam because I had to work while studying. It was really difficult… because I had a family…. and I had to earn money so it was really hard”* (IMG 10). It took him two more years to pass the test and continue with the internship. During this time, he felt frustrated and thought about giving up. Another IMG had to leave her family and 2-year-old son during the weeks throughout the supplementary training. She had received support from her husband, mother, and mother-in-law, but described how she had missed spending time with her son. Some IMGs thought the supplementary training should involve more information about cultural differences, which were described as unwritten rules. One IMG reported that you can get help from colleagues, but you have to ask for it; no one will offer it to you.

For IMGs with a specialization, the process was shorter, but they also had some difficulties in getting their clinical training. For all IMGs, the requirements for getting a license when coming from outside the EU were perceived as unfair compared to the requirements for IMGs coming from the EU. They mentioned having felt left out from working life and having forgotten medical knowhow because this process took a few to several years.

##### The work is familiar, yet a lot is new

This theme concerns the experience of starting work at a new workplace in a new country. It involves the IENs’ and IMGs’ perceptions about how their new employer welcomed them, what differences they found in the host country compared to their earlier work experiences, but also feelings of being competent as a nurse or a doctor. Furthermore, it concerns how they used their intercultural competence at the workplace.

#### Being introduced

The IENs reported feeling welcome at the new workplace. They said their new colleagues were open toward them, kind and showed interest. Most IENs reported not feeling like an immigrant or being offended at work: “*I don’t feel like an immigrant when I’m with them”* (Auxiliary nurse, 2). Concerning their education, the IENs sometimes felt that their Swedish colleagues questioned it. They described this as annoying, because they felt their education was just as good as Swedish education, and to some extent better. A few said being an immigrant was not easy: “*Well, maybe someone didn’t show the same respect to me as to a Swedish colleague. Maybe they have more respect for a Swede”* (IEN 8).

In general, the IMGs reported feeling welcome at work and that their colleagues were kind to them. One IMG, with an education from outside the EU, described the fantastic feeling when he finally started working and could talk to doctors and nurses on the ward. Furthermore, the feeling of being a member of the team was important, as was the feeling of being accepted by co-workers: “*And no, I think they don’t treat me in any way other than team members here. I feel like home. I am home”* (IMG 9).

The IMGs doing their internship went to many workplaces and could describe feeling welcome at some workplaces and unwelcome at others. Those who underwent an internship said that it was good to get the same introduction at each workplace as the Swedish doctors. A few IMGs thought it was difficult to know what was expected of them during the internship and how much responsibility they had.

A few IMGs mentioned situations in which they had felt offended in various ways. Some reported disagreement and unfriendliness between doctors educated in different countries. A few mentioned that colleagues had been mean to them, and one specialist felt unwelcome in the operating room. A few reported that conflicts or misunderstandings with colleagues arose due to different working procedures. Those who had experienced difficult situations or unfair treatment described how they felt sad and angry, and one had questioned whether it was possible to work in Sweden: “*… there were periods when I wanted to leave Sweden. I thought maybe I wouldn’t be able to work here. [but] I do now”* (IMG 8). To overcome the negative experiences, some had talked to their supervisors and actions had been taken at the workplaces. One had contacted the occupational health service to get support. However, some decided just to keep working without thinking too much, and not to let the negative incidents continue to influence them: “*Because I can’t change my origins, I can’t change such things, so I’ve decided not to think about them”* (IMG7). A few IMGs reported having received less pay at the beginning compared to Swedish physicians. Feeling welcome as a member of a working team was important, therefore IMGs who could, changed jobs until they found a workplace where their colleagues respected them.

#### Observing differences and adjusting to them

The IENs were ambivalent when they compared work in Sweden to work in their home country. They described differences, but at the same time reasoned that these differences were minor. The technical aspects of nursing work were almost the same everywhere, while many routines were new. In general, the IENs reported that the use of technology was more common in Sweden than in their home countries, and some felt that nursing in Sweden had a strong focus on medical aspects of care. Some IENs were not familiar with using technical monitoring equipment, for example on the gynecology ward, and some were accustomed to using natural medicine, which they said was uncommon in Sweden. Furthermore, they had to spend more time now on documentation in the medical records, and some mentioned that interaction with patients was more central than they were used to.

Overall, the main differences for the IENs were related to work culture and the healthcare system. As nurses, they felt more appreciated at work and had more responsibility in Sweden than in their home countries. However, one midwife mentioned that she could work more independently in her home country. Although the IENs reported having adjusted to Swedish working methods because they had moved here, it was not always so easy. Therefore, they sometimes wanted to use their own methods, but other nursing staff did not appreciate such initiatives.

The IMGs described both similarities and differences when comparing their work in Sweden to work in their home countries. According to many, medical work was the same no matter where doctors practice. For example, the recommendations for treating childhood asthma were the same in Europe, but the drug names were new, and that took time to learn. Regarding differences, many mentioned that the healthcare system in Sweden was different and something one had to get familiar with. This concerned learning to write a medical certificate, learning about sick-leave rules, treatment recommendations for various health problems from the SNBH, the restrictive use of antibiotics and the need to call a specialist when working in a hospital instead of treating the patient yourself. Some IMGs had not made dictations before and some had not used computer-based medical records, and they said it took one to two months to learn this. They found documentation very time consuming. Compared to their home countries, in Sweden they had fewer patients each day and wrote more in the patient/medical records. A few mentioned that Sweden was less hierarchical and that they appreciated working in teams with people from other professions. Listening to everyone’s opinions was appreciated: “*There aren’t these levels like… hierarchies like in other places, where I come from for example. There it’s really pronounced, the chief physicians are above everyone, they make all the decisions, and you just have to accept them. But here everything and everyone are taken into consideration… the assistant nurses and … and everyone is part of the same team and listens to each other. And I think that’s really positive”* (IMG 10). Regarding the communication with patients, some mentioned that patients in Sweden had more knowledge about their illnesses and thus it was easier to talk with them, while others felt they as physicians had the most knowledge and therefore should have more say.

Both IENs and IMGs would have appreciated having more time to learn about Swedish culture and healthcare system at the beginning.

#### Feeling competent

All IENs had undergone some introduction at their first workplace, varying from one to six months. Overall, the IENs felt they were competent in nursing, even though there were situations in which they felt unsure. They felt they had medical and nursing knowledge, but that they had to learn the new routines at the workplace: “*The practical knowledge I knew, but the routines here on the ward you have to learn and keep learning your whole life”* (IEN 1). Some IENs reported having unexpected responsibilities as registered nurses; for instance, being in charge in the event of a fire outbreak at night was an unwanted task.

In general, the IMGs had shorter workplace introductions compared to the IENs, varying from one day to four months. Some IMGs described very good supervisors who had time to introduce them to their job and new working routines. In these cases, they had had fewer patients each day and “easier” cases than the other doctors at work. They further mentioned that their colleagues were understanding and that they took on greater responsibility as they learned more. In other situations, some IMGs reported having started work with a very brief or minimal introduction. The supervisors they had were difficult to get in touch with if they had questions: “*What was really hard was that I was thrown into medical care without having received any real guidance… I mean at first I thought I’d work beside someone for a while, to get oriented to how people work and think. But I didn’t get to, I was somehow thrown into the work straight away”* (IMG7). In general, the IMGs felt they had the right qualifications for working in Sweden. However, their profession as medical doctors required lifelong learning, and they pointed out the need for further training.

#### Using intercultural competence and life experiences

The IENs felt that their colleagues were interested in hearing about their country or culture. They reported that experiences from other countries and cultures were very useful at work. The IENs could speak different languages and were sometimes asked to help with translations. They also felt they were able to understand the customs and beliefs of patients from other countries. This could concern food culture, religion or just how people express themselves. A few IENs mentioned that they could understand and interact with patients from other countries better because they themselves had come to Sweden as immigrants: “*…. because I also came here as a refugee and well, I shouldn’t say I understand, but I’ve been in their shoes and then you have another feeling for things”* (IEN 8).

Among the IMGs, the responses varied regarding using one’s intercultural competence at work. A few IMGs said they tried to leave the culture from their home country behind them and wanted to adjust to Swedish culture: “*I try to adapt to the culture here. Not bring my culture here. Because I came to Sweden, I’m Swedish. …. So I can’t really have my culture, have it here”* (IMG 9).

Others mentioned having tried to explain cultural differences to patients. One IMG described how he tried to convince patients from his home country to undergo surgery, explaining that the procedure was less risky in Sweden. Another mentioned how he had to explain the restrictive use of antibiotics, and yet another had tried to explain to immigrants how the Swedish healthcare system works. All said that they were willing to use their native language or other language skills in encounters with patients. Some used other languages than Swedish several times a week with immigrant patients. They were also asked by colleagues to translate for them. They experienced that talking with patients in their own language increased patients’ satisfaction.

##### Trying to master a new language

This theme concerns the importance of mastering a new language and how to learn it. It also concerns how lack of language skills impacts work performance and inclusion at work. Here the professions are presented together, as what they had to say was very similar.

#### Learning a language in various ways

Although all IENs and IMGs had to learn Swedish, they had very different experiences of the learning process. Some IENs and IMGs from the EU had started working without any formal education in Swedish; they learned the language on their own and at the workplace, and one IMG was still studying Swedish to pass the new language requirements. One IEN and two IMGs who had been recruited were offered a language course before coming to Sweden. Nurses and physicians coming from outside the EU had taken several courses and started studying Swedish for immigrants, and thereafter continued taking courses until they passed the language tests. Learning Swedish in this way took about two years: “*You could say I studied for two years, language for two years, and during that time I didn’t work as a doctor, didn’t study medicine, so I was kind of out of it”* (IMG 4). One IMG had asked whether he could participate in an intensive language course, but was told it was not available. Another IMG was able to visit a health clinic during the language course to practice language skills. A few IENs and IMGs had studied a language course designed for healthcare staff and reported that the course had helped them a lot. The IENs and IMGs who took the supplementary training said that the course had facilitated language learning, and some mentioned that colleagues had been supportive in helping them learn Swedish.

#### Feeling insecure and inferior in relation to limited language skills

Poor language skills created negative feelings among IMGs, and some IENs felt insecure at work. Some thought that patients could not trust them fully because of their insufficient language skills: “*You feel a bit inferior when you don’t know the language”* (IEN 9). One IMG described her first meeting with a patient as “terrible” because she could not speak so much with the patient, and a nurse had stepped in and helped with the language. Another IMG felt like an outsider even though colleagues were nice and tried to talk. The same IMG mentioned that it was difficult to actively participate in discussions because of differences in the expressions used by IMGs and native physicians. Insufficient language skills caused IENs and IMGs to avoid discussions in the workplace.

#### Language skills – The main problem

All IENs and IMGs mentioned the importance of learning the native language in order to work. It was described as the main problem for IENs and IMGs. The specific terminology in medicine and nursing was difficult, and the challenge was to make use of their existing knowledge in a new language: “*You have quite a bit of medical knowledge, it’s not the nursing part, that’s not the problem but instead transferring it to a completely different language, that’s an art. Language is really important and if it takes too long the risk is you’ll give up”* (IEN 10). One IMG stated that to work, you have to be able to speak Swedish very well. Some IENs and IMGs described feelings of stress at work due to limited language skills, and a few IMGs mentioned that it was difficult to speak with immigrant patients because both of them had limited language skills. The first two months were the most difficult time for IENs and IMGs, thereafter it became easier to talk with colleagues and patients. However, having insufficient language skills for longer periods could be problematic. One IMG said he had major problems with the language the first two years at work. Regarding socialization in the workplace, both IENs and IMGs said it was hard to communicate during coffee breaks when colleagues talked everyday Swedish in the staff room: “*There is a lot of stress at first. A lot. You’re all wound up all the time... You understand patients and the medical things, that’s what you learn quickest. But then there are the coffee breaks and people are talking and you have an idea what they’re talking about but you don’t really know what they’re saying”* (IMG 7).

## Discussion

The present study aimed to explore the experiences of IENs and IMGs when applying for a license to practice and work in the Swedish health and social care system. It showed that some of the experiences of getting a license to practice one’s profession and the experiences of entering a new workplace in another country were the same for nurses and physicians. However, nuances in their experiences were found. Regarding the workplace introduction, IENs reported longer introduction times than did IMGs. In general, both IENs and IMGs in the study felt welcome at work, but the IMGs reported more instances of disagreement between colleagues, and some had experienced discrimination. Both IENs and IMGs used their intercultural competence at work. Lack of language skills was described as the main problem for both IENs and IMGs. Insufficient language skills among IENs and IMGs created experiences of stress, difficulties in socializing at work and feelings of inferiority. The results can be compared with a review study from the UK [36] that shows that cultural awareness, discrimination exposure, language skills, and formal and informal networks were central to integration experiences. However, in the review they found indications that nurses experienced more deficiencies in knowledge and skills as well as more discrimination than doctors did. The hierarchical environment was discussed as one possible explanation. In the present study, however, the healthcare environment was described as less hierarchical compared to that in the participants’ home countries.

IENs and IMGs from the EU reported no major obstacles to obtaining a license to practice their profession in Sweden. However, IENs and IMGs from countries outside the EU described various barriers to obtaining a license. Some IENs had difficulties getting information from the SNBH about how to apply for a nursing license. Licensing barriers for IENs seeking job opportunities in other countries have previously been reported by Moyce et al. [8]. Both IENs and IMGs described financial problems when they had to work during their supplemental training, even if student aid, including both grants and loans, is available for students with a residence permit in Sweden. The result is in line with a study from the Netherlands [17], where lack of financial support was one of the barriers for IMGs; they were allowed to begin supplementary medical education, but were not offered any financial support. Furthermore, the IMGs felt left out of working life, because it took them a long time to learn Swedish, finish the supplementary education and obtain the license to practice medicine. Similar findings have been reported from Canada [6] and the UK [15], where IMGs experienced a loss of professional identity in the process of acclimatizing to the host country.

Our results showed that IENs and IMGs from non-EU countries had more difficulties obtaining a license and achieving successful integration into the workplace (cf. Davda et al., [36], who found long registration processes to be the main integration barrier). This may be related to the time it takes for this group to complete their supplementary training and language courses. In comparison, IENS and IMGs from within the EU only have to prove their language skills and can obtain their license after the administration time has passed. This raises questions about how this process could be speeded up. The importance of tailored support programs is emphasized here and in other studies [36, 37]. Initiatives in Sweden, the so-called “fast track,” are trying to help immigrants with a university education, including IENs and IMGs, find employment. This may involve intensive language education and offering learning opportunities, for example by visiting a health clinic over a period of time. However, whether these initiatives help speed up the process of obtaining a license has not been evaluated. Strategies intended to promote successful integration of IENs and IMGs should, therefore, focus specifically on people coming from outside the EU. Further, information about the application process, the required supplementary training and the support available during this process should be made more accessible. Covell et al. [37] found bridging programs to be a predictor for finding the first job in the new country. Predictors for passing the licensure exam were working experience (3–5 years) and help studying for the exam.

Our results showed that, overall, both IENs and IMGs felt welcome at their new workplace. However, some IENs had experienced colleagues questioning their international education. Further, IMGs described how differences in their medical education had created conflicts with colleagues. In addition, a few IMGs in the present study expressed feelings of being offended in various ways. This is in line with previous studies [8, 17, 29, 38] reporting how both IENs and IMGs experience workplace discrimination. Discrimination, in any form, needs to be addressed. However, arguing, for example, that someone has insufficient knowledge need not be an expression of discrimination, but could be perceived as such, depending on the person and how the argument is made. This is sometimes a problem with physicians from other countries, where for example limited language skills also influence the performance of IMGs. Managers and organizations employing internationally educated healthcare professionals have to be aware that discrimination can occur and work proactively to prevent it.

Concerning cultural differences, IENs and IMGs reported that technical aspects of nursing and medical work were similar in Sweden and their home countries. However, in Sweden, they spent more time documenting patient/medical records than they were used to. Furthermore, both IENs and IMGs described how they had to learn about the Swedish healthcare system. The challenge of adapting to a another healthcare system has been widely documented [8, 18, 24]. For IMGs this could involve, among other things, following national treatment recommendations for various diseases, knowing the sick-leave rules and writing various certificates. For the IENs, this could involve adjusting to a different working culture with greater responsibility for patient care than they were used to, a finding also reported by others [23]. Some IENs reported wanting to apply some of their old working methods in Sweden, but that their colleagues did not appreciate such initiatives. This indicates that the acclimatization process takes time, as the IENs had longer introduction periods than the IMGs did. The problems of language and culture are often underestimated, and these, of course, are very similar for doctors and nurses. Therefore, supplementary training for non-EU IENs and IMGs should more clearly include topics about culture and how the host country’s healthcare system works. There should also be more opportunities to discuss and reflect on differences and similarities between routines and the healthcare systems with colleagues/mentors (cf. [15]).

In the present study, workplace introduction for IMGs was relatively short. This result is consistent with a realist synthesis [39], where time and limited resources were reported to hinder comprehensive interventions to facilitate transitions for IMGs. The same study showed that IMGs prefer a longer workplace orientation to facilitate the acculturation process. Moreover, a Finnish study [40] found that IMGs, compared with native Finns, more often experienced lack of professional support. It has been suggested in the literature that offering regular learning opportunities for international staff leads to more effective acclimatization in the workplace [15]. Furthermore, it has been reported that introduction programs for IMGs need to be more in line with the needs of IMGs and specific to the new country [39, 41]. This indicates that workplace introduction for IMGs could be improved by offering them longer and more organized introduction programs.

Regarding competence, our results showed that IENs and IMGs generally felt competent at work. A review from the UK [36] reported knowledge gaps among nurses, but not among doctors. However, it also suggested that a change might have occurred after competency tests were introduced in the UK in 2014 for non-EU nurses similar to those for non-EU doctors. Although, the IENs and IMGs experienced problems related to moving to another country (language difficulties, adapting to a new culture and healthcare system), they expressed feelings of competency in their profession. According to Bandura [42], perceived self-efficacy refers to individuals’ belief in their own effectiveness regarding coping with a given situation. The IENs and IMGs in the present study expressed strong self-efficacy in relation to the work itself. However, earlier knowledge needs to be integrated with knowledge about the host country’s healthcare system and routines.

A Norwegian study [43] found that IMGs were reluctant to ask colleagues for support because they wanted to be seen as competent. The problem of lacking knowledge while needing to maintain one’s image at work may be problematic. Thus, even if IENs and IMGs feel competent in the work itself, the surrounding organization is new and difficult to deal with. Swedish colleagues could be more open and ask IENs and IMGs if they need some guidance in understanding the Swedish healthcare system. Perceived self-efficacy, feelings of competency and advice on how to integrate previous knowledge into a new healthcare system should be explored in more detail in future studies. In addition, because the present study did not explore the different practical training educational experiences of IENs and IMGs, this is another important issue for future research.

The majority of IENs and IMGs used their intercultural competence at work. Especially the IMGs stated that talking with patients in their own language increased patients’ satisfaction and self-esteem. Furthermore, the IMGs reported explaining cultural differences to immigrant patients. This result raises the question of whether it is an advantage for immigrants in Sweden to see a physician or nurse with a foreign background. McKimm and Wilkinson [22] stated that making cultural transitions is complex for IMGs, and concluded that those from other cultures help to make today’s multi-cultural health services more diverse, which is clearly needed. Future research should focus on how patients and their relatives experience the intercultural competence of both native and international staff.

Regarding language, data collection took place both prior to and after the SNBH introduced Swedish language requirements in 2016. Therefore, the participants in the present study described different experiences concerning how they learned Swedish. However, regardless of how they learned Swedish, it took a long time for both IENs and IMGs to learn the language. In the study, IENs with poor language skills reported feeling inferior when interacting with patients and colleagues. This result is consistent with previous research. An integrated review [23] found that, because of language difficulties, IENs experienced feelings of deprecation and low self-esteem. In the present study, both IENs and IMGs described how lack of language skills hindered interaction with colleagues and patients, which has also been reported by others [23, 43]. This may be an even greater problem when learning a completely new language like Swedish as compared to English, which many people learn during school. Language problems could be one explanation for the negative association found between hospitals with a higher percentage of IENs and lower patient satisfaction [44]. Being able to communicate efficiently may be of even greater importance in healthcare settings that focus on person-centered care and shared decision-making. Mastering a new language was further described as the main problem for IENs and IMGs, as pointed out in several studies [8, 13, 17–19, 23]. This indicates that language courses specifically aimed at healthcare staff need to be more accessible. In Australia, a language and communication skills web resource for English has been developed for doctors with a non-English-speaking background, and the website has been popular [16]. It is possible that a similar resource for Swedish could be helpful for IMGs coming to Sweden, and a similar program could be developed for IENs. A Dutch study found that offering additional employer-funded language courses was a facilitating factor in improving language skills among IMGs [17]. This indicates that employers need to facilitate learning opportunities for IENs and IMGs to develop their language skills, in this way helping them acclimatize more efficiently.

More studies on the experiences of IENs and IMGs in other European countries are needed. Such studies would benefit from using a larger sample size to explore the needs of IENs and IMGs who are seeking a license to practice in a foreign country. Additionally, comparisons should be drawn between the experience and needs of immigrants from within the EU and those from outside the EU. Studies examining workplace introduction programs, especially for IMGs, could help us better understand how these programs could be improved to better meet the needs of IMGs.

### Strengths and limitations

To strengthen credibility, IENs and IMGs who varied in terms of home country, age, and work experience in Sweden were included in the study. However, two midwives worked as auxiliary nurses and one physician had not passed the language test, which reduced the number of responses to some questions. On the other hand, this is also a strength, as these participants were in the middle of the process of getting a license. The informants were asked whether they wanted to perform the interview in Swedish or English, and they clearly stated that they wanted the interview to be in Swedish. This led to some variation in their answers to some questions due to limited mastery of Swedish. To enhance transferability, a detailed description of the participants’ demographic characteristics has been presented, as well as details about data collection and the steps in the analysis. However, the present study is based on a small sample size from a Swedish context, and transferring the results to other contexts must therefore be done with caution.

## Conclusions

The present study confirms findings in earlier, mostly English speaking countries, in regards to language difficulties being the main problem for IENs and IMGs. The importance of adequate language skills has not previously been a focus in Sweden. This study, however, underlines the importance of offering better language training as an essential part of improving workplace integration in Sweden. The study presents new knowledge, as the IENs and IMGs reported facing quite similar challenges in the host country. The differences found mainly related to whether the IENs and IMGs came from within the EU or outside the EU. Furthermore, the findings confirm earlier research regarding the importance of the interplay between IENs and IMGs and the social context in the new country. Workplace introduction was shorter for IMGs than for IENs. Employers should provide longer and regular learning opportunities, especially for IMGs. Further, more time-efficient language courses should be offered to IENs and IMGs. Online language courses, specifically developed for IENs and IMGs, could be useful, as could offering language education more formally at the workplace. Both IENs and IMGs used their intercultural competence at work. Future studies should explore whether immigrants seeking care in Sweden would benefit from seeing a multicultural health team.

## Additional file

Additional file 1: Interview questions to IENs and IMGs. (DOCX 17 kb)

## Abbreviations

IEN: International Educated Nurses
IMG: International Medical Graduates
SNBH: Swedish National Board of Health and Welfare
