# Supplementary material for: Internationally educated nurses’ and medical graduates’ experiences of getting a license and practicing in Sweden – a qualitative interview study
Source: BMC Med Educ. 2018 Dec 5;18:296. doi: 10.1186/s12909-018-1399-4 (PMC6282362; doi:10.1186/s12909-018-1399-4)
Supplement: Supplementary file 1 — Interview questions to IENs and IMGs. (DOCX 17 kb) [file 12909_2018_1399_MOESM1_ESM.docx]

Additional file 1. Interview questions to IENs and IMGs

| Interview Questions: |
| --- |
| Sex |
| Age |
| Period of education (years) |
| Country of education |
| Number of years working as a nurse/physician |
| Number of years working as a nurse/physician in Sweden |
| Tell about your first period of time as a nurse/physician in Sweden, beginning with the first day – what was it like? Your first month? |
| How would you describe your experiences of working as a nurse/physician in Sweden?  What are your most positive experiences of working as a nurse/physician in Sweden?  Do you have any negative experiences of working as a nurse/physician in Sweden? |
| If you consider the country in which you were educated/worked previously, what are the greatest differences/similarities compared with working as a nurse/physician in Sweden?  Can you describe how you have dealt with these differences? |
| What introductory program have you received to help you adapt your working methods to conditions in Sweden? |
| Can you describe what it is like to become part of the community at your workplace? |
| Have you noticed any conflicts between nurses/physicians with a foreign education and Swedish nurses/physicians, or between nurses/physicians from different cultures? |
| If you consider well-being in relation to work, talk about how you feel at work. |
| Various factors can cause a person to experience work as trying or stressful; can you talk about what factors contribute to making you experience work as stressful? |
| What is it that makes you happy/not happy at work? |
| Tell about how you experience your work environment. |
| How do you experience the physical conditions at your workplace? |
| How would you describe the work atmosphere at your workplace? |
| How do you view your work in terms of its meaningfulness? |
| How do you view your competence as regards performing your work tasks? |
| How do you view your ability to influence strategic or administrative decisions at your workplace? |
| What would you like your work situation to look like in five years? |
| How do you make use of your cultural competence in encounters with patients? |
| How do you make use of your cultural competence in encounters with other health and medical care staff? |
| How would you like to use your cultural competence? |
| Do you feel your cultural background and experience are taken advantage of at your workplace? |
| If you were part of a working group tasked with designing the ideal introduction program for nurses/physicians who come to Sweden, what would that program look like? |
| Is there anything you would like to add that was not covered by the questions raised here? |
